# Caring for and Caring about in Economic Evaluation: Modelling the Family and Caregiving Effects

**DOI:** 10.1007/s40273-025-01540-w

**Published:** 2025-11-01

**Authors:** Becky Pennington, Sarah Davis, Holly Cranmer

**Affiliations:** 1 Sheffield Centre for Health and Related Research, School of Medicine and Population Health, University of Sheffield, Sheffield, UK; 2 Cranmer Consultancy Ltd, Sheffield, UK

## Abstract

Current methods for modelling spillover effects on carers in economic evaluation include four main methods: (absolute) utilities, disutilities, increments and multipliers. Each of these approaches assumes that the spillover effect is one-dimensional. We aimed to develop a new approach that better reflects the complexity of caring and the nuances of how a new treatment may impact the caregiver. We propose a new method based on the established concepts of the ‘family effect’ (or caring about someone) and the ‘caregiving effect’ (providing care for someone). These effects can be disentangled through analysis of carer-patient dyads or using patient and carer (dis)utilities and estimates from the literature. We consider case studies in Duchenne Muscular Dystrophy, Spinal Muscular Atrophy and Alzheimer’s Disease. Our approach models a small carer health-related quality of life (HRQoL) gain for each intervention, whereas the utility approach consistently models a substantial carer HRQoL gain, and the disutility approach models a carer HRQoL loss in two case studies. Our method allows explicit consideration of the benefits to carers of extending patient survival or improving patient health, with the negative HRQoL impact of increased caregiving burden. We propose that our method can be used with published data at present, and further research should analyse the family and caregiving effects in different conditions.

## Key Points for Decision Makers

**Table:** 

Previous research has disentangled caring into the family effect (caring about someone) and the caregiving effect (caring for someone). We demonstrate that numerical estimates can be attributed to these concepts to allow them to be used to model carers’ health-related quality of life in economic evaluation.
Modelling the family and the caregiving effects allows a trade-off between the benefits to carers of extending patient survival against the negative health-related quality of life impact of increased caregiving burden. Traditional modelling approaches have assumed caring is either wholly negative or wholly positive.
In three case studies where interventions improve morbidity and patient survival, modelling the family effect and caregiving effect predicts a small impact on carer health-related quality of life.

## Current Methods for Modelling Spillovers on Carers

In cost–utility analyses, the cost-effectiveness of a new treatment is assessed based on the cost per additional quality adjusted life year (QALY) gained, where a QALY is calculated from patient life years multiplied by utilities. Typically, these analyses include only patient utilities. However, as there is increasing recognition for the wider impact of therapies, some cost–utility analyses may include spillover effects for carers/caregivers (hereafter, carers), to reflect the impact of treatment on carers’ health-related quality of life (HRQoL).

The literature highlights different approaches to modelling carers’ HRQoL in economic models [1–3], we refer to these throughout as:

1. The ‘utility’ approach (also known as the ‘absolute utility’ or ‘additive’ approach), which models carers’ utilities based on the patients’ health states and QALYs are calculated separately for patient and carers and summed.
2. The ‘disutility’ (or ‘utility decrement’) approach, which models carers’ disutilities (calculated as the difference between full health or age-adjusted population utilities and the carers’ utility), based on the patients’ health states.
3. The ‘increments’ approach, which includes the ‘incremental effect’, or difference between carer utilities relative to some baseline health state.
4. The ‘multiplier’ approach, which estimates carer QALY gains as a proportion of patient QALY gains, using a ratio.

The first three approaches attach a specific carer HRQoL value (a utility, disutility, or change in utility) to each patient health state and multiply this by either the total or incremental patient life years in the specific health state. This is repeated for each health state, and they are summed to give the total carer QALYs or QALY losses. For the utility approach, this carer HRQoL value is positive. For the disutility approach, this carer HRQoL value is negative. The carer HRQoL may be positive or negative when using the increments approach depending on the relative difference in carer utilities across health states and the baseline health state used in calculations (negative values, for example when calculated relative to the best health state may be termed ‘decrements’). The multiplier approach instead applies a ratio or ‘multiplier’ to the total patient QALYs and does not involve consideration of individual health states, nor does it require availability of caregiver utilities.

The disutility approach has been most commonly used in technology appraisals and highly specialised technology evaluations for the National Institute for Health and Care Excellence (NICE) in England [4, 5], with the utility approach used in some cases [1, 4] and the increments approach more recently [6–8]. The utility approach has been used by evaluations undertaken in the Netherlands and Sweden [1]. An important criticism of the disutility approach is that it implies that interventions that improve patient survival led to a QALY loss for carers, because the negative impact of caregiving is extended. This has been termed the ‘carer QALY trap’ [9] and has been critiqued as incongruent with altruism [10], and the importance of considering altruism in spillovers is long-established [11]. The utility approach has been critiqued as devaluing bereaved caregivers, since their utility is assumed to be zero after the patient dies [12].

The multiplier approach has been less commonly used in health technology assessment but has been more broadly used in economic evaluation [13]. A case study in meningitis used a multiplier of 0.16 [14] derived from a regression model to estimate family members’ EQ-5D-5L as a function of patient EQ-5D-5L [15]. The authors note that if multipliers are constant across interventions, then decision-making based on patient health benefits alone is sufficient to maximise population health benefits. However, it seems unlikely that multipliers are constant across interventions given that interventions have the potential to affect carers and family members via multiple different mechanisms [16]. The Dental and Pharmaceutical Benefits Agency in Sweden recently discussed the multiplier method and proposed a similar method, in which carers are assumed to gain a proportion (using a ‘standard rate’) of the patient’s QALY gain during the period the patient would have lived with standard care. However, they note that this is not based on actual measurements and the magnitude of the standard rate needs to be determined [17].

The utility approach and multiplier method both assume that (where the multiplier and carer utilities are positive), interventions that improve patient QALYs (by improving patient HRQoL and/or extending patient survival) necessarily lead to a QALY gain for carers, and therefore assume that the HRQoL impact of caring is entirely positive. The disutility approach assumes that interventions that improve patient HRQoL without affecting survival improve carer HRQoL, but interventions that extend patient survival lead to a HRQoL loss for carers, therefore assuming that the HRQoL impact of caring is entirely negative. The increments approach also assumes that the HRQoL impact of caring is one-dimensional, but the direction of impact depends on the baseline health state.

In reality, caring is complex, and the effect of caring on carers’ HRQoL can have positive and negative aspects [18]. We aimed to develop a new approach for modelling carers’ HRQoL that better reflects the complexity of care and allows for simultaneous positive and negative impacts.

## How does Caring Affect Carers’ HRQoL?

### Family and Caregiving Effects

To model changes in carers’ HRQoL in economic evaluation, it is important to first understand what affects carers’ HRQoL. In health economics, the effect of caring on carers’ HRQoL has been analysed as a two-part process: caring about, termed the ‘family effect’ (equivalent to the first phase of care ‘caring about’ proposed by Fisher and Tronto [19]) and caring for, termed the ‘caregiving effect’ (equivalent to Fisher and Tronto’s second and third phases: ‘caring for’ and ‘caregiving’). The family effect may be interpreted as the emotional impact of a loved one’s illness (which may persist even for people who are not directly involved in providing care), whereas the caregiving effect reflects the burden or practical impact experienced by those actively engaged in caregiving. These two effects can be easily captured by measuring the health, HRQoL or wellbeing of the ‘other’, and by recording the caregiving tasks performed.

Bobinac et al. (2010 and 2011) used cross-sectional analysis on carer and care-recipient dyads to disentangle the family effect and caregiving effect on HRQoL [20, 21]. They used regression analyses to estimate the carers’ wellbeing (2010) or health (2011), as a function of the patient’s HRQoL (2010) or health (2011) to represent the family effect, and the number of caregiving tasks (2010 and 2011) to represent the caregiving effect, as well as demographic variables. Bobinac et al. (2011) found a significant correlation between patient and carers’ health of 0.13, indicating that carer’s health improved by 0.013 as patient’s health improved by 0.1 (both measured using EuroQoL-VAS) [21]. Bobinac et al. [20] found a smaller but significant correlation of 0.0642, between carer’s wellbeing (measured using a self-report happiness scale) and patient HRQoL (measured using EQ-5D). The correlation between patient and carers’ health/HRQoL/wellbeing may be interpreted as a ‘family effect multiplier’, since it is multiplied by the patients’ HRQoL to give the family effect on the carers’ HRQoL. In both studies, carers’ HRQoL significantly decreased as the number of caregiving tasks increased (the caregiving effect).

Pennington et al. used fixed-effects analysis on 13 waves of longitudinal data for carer and care-recipient dyads to identify family and caregiving effects. The family effect, represented by the correlation between changes in patients’ and carers’ HRQoL using SF-6D (the family effect multiplier) was 0.123. The caregiving effect was captured by measuring the relationship between intensity and duration of caregiving with carers’ HRQoL [22]. Their analysis used a UK survey that included patients with a range of health conditions and found that the family effect multiplier was similar when the patient did or did not report having an illness. The caregiving effect was slightly more complicated: each additional year of care decreased SF-6D by 0.045 and authors advised caution against extrapolating over a long period since relatively few carers cared for more than 5 years. Caregiving intensity had a positive impact on carers’ HRQoL but this was quickly negated by the negative effect of duration of care. However, caregiving at low intensity (0–9 h/week) had a smaller positive effect (0.032 for SF-6D) than at medium (10-19 h/week, 0.077 for SF-6D) or high intensity (20+ h/week, 0.058 for SF-6D). This suggests that, assuming care-recipients had the same HRQoL and had received care for the same duration, people caring for fewer hours per week would have lower HRQoL than people caring for more hours.

### Using the Family Effect and the Caregiving Effect to Estimate Spillover Effects in Economic Evaluation

The family effect has generally been demonstrated to be positive [20–22] which aligns with qualitative evidence that treatment of patients’ symptoms was linked to improved carers’ emotional health, and that family carers valued improvements in patients’ independence and skills [16]. We therefore expect that as patients’ HRQoL improves, carers’ HRQoL improves (all else being equal). The caregiving effect may be negative where providing a higher volume of care leads to worse carers’ HRQoL, or it may be positive in the scenario that, after adjusting for the family effect, the act of providing care leads to a gain in HRQoL (as suggested by Han et al 2021 [23] and Han 2023 [24]).

The family and caregiving effects both contribute to the carers’ utility, but they do not fully explain the carers’ utility since HRQoL is also affected by other factors (such as age, sex, family set up and income [22]). The carer’s utility may therefore be thought of as the sum of these other factors, plus the family effect (calculated by multiplying the family effect multiplier by the patient’s utility), and the caregiving effect. The carer’s disutility, or difference between the carers’ utility and the expected utility for someone who is not caring is therefore due to the family effect of the patient’s ill health (calculated by multiplying the family effect multiplier by the patient’s disutility) and the caregiving effect.

Using our approach, we estimate the impact of carer QALYs by multiplying the time a patient spends in each patient health state by the sum of the family and caregiving effects for that health state. This is different from multiplying the time in the health state by the carer’s utility, since we do not include the part of utility that is unrelated to caring. It is also different from using the carer’s disutility, since we consider the positive family effect of the patients’ utility, rather than the negative family effect of the patient’s disutility. Disentangling the family and caregiving effects in this way enables recognition that some patient interventions may affect the HRQoL of the patient and not the caregiving burden or may affect the caregiving burden and not patient HRQoL (for example, substituting unpaid care with formal care), or may affect both.

### Patient Death and Bereavement

The utility approach and multiplier approaches may be interpreted as implying that carers’ HRQoL becomes equivalent to death (0) after the patient dies. The disutility approach assumes that any negative HRQoL impact is lost as soon as the patient dies. The implication of the increments approach depends on the relative baseline. The inclusion of an explicit HRQoL effect of bereavement on deceased caregiver’s HRQoL has been proposed as a solution to the carer QALY trap [25], but there is little evidence to inform its inclusion [26]. It is therefore important to consider the implications of the family and caregiver effects after patient death. It seems intuitive that when the patient dies the caregiving effect becomes zero (since there is no longer a caregiving requirement), but the family effect, which had previously been a positive effect must also become zero since the utility for the dead patient is zero (and any effect multiplied by zero is zero). So, if the family effect is greater than the caregiving effect, extending patient survival will typically result in a QALY gain for carers. The bereavement effect is not explicitly modelled, but premature patient death will result in carer QALY gain forgone, the magnitude of which depends on the what the family effect and caregiving effect would have been had the patient survived, and the difference in duration of patient survival.

### Data Sources

Ideally, analyses of carer utilities will be able to provide both the family and caregiving effects, but it is more common to simply have carer utilities, or carer disutilities. In this case, these values may be presented as a caregiving effect but already capture the family effect. In this scenario, we need to first estimate the family effect using the multiplier and the patient utilities and then calculate the caregiving effect as the remaining disutility. Where we do not have estimates of the family effect multiplier for that population, we will have to use family effect multipliers from other analyses. It is possible that the family effect multiplier varies across different populations, but quantitative evidence for this is limited. The multipliers discussed above are fairly similar (0.16 [14], 0.13 [21] and 0.123 [2]), despite coming from different populations. This may suggest that it is reasonable to assume that the family effect multiplier is transferable across populations. However, a study on the effect of spillovers of childhood conditions on maternal depression found that while childhood illness that severely hampered daily activities was associated with a statistically significant 0.12 increase in the probability of maternal depression, only some specific childhood conditions were associated with maternal depression, with particularly large effect sizes of mental/behavioural conditions [27]. In a study focussed on spillover effects of autism in children, caregiver’s HRQoL scores differed in relation to child health or behaviours, suggesting that the determinants of carer HRQoL spillovers vary across different domains [28]. It is unclear whether these domains would affect care-recipient and caregiver HRQoL proportionately (in which cases multipliers would be constant) or be captured through measures of the caregiving effect, or whether multipliers vary across populations.

## Case Studies

### Selected Case Studies

We consider the application of the family and caregiver effects in three real-world examples using published information on discounted patient life years, patient utilities and carer (dis)utilities in each health state. We were able to access this information in the company submissions for two NICE Highly Specialised Technology appraisals: ataluren for treating Duchenne muscular dystrophy (DMD) with a nonsense mutation in the dystrophin gene [29] and onasemnogene abeparvovec for treating spinal muscular atrophy (SMA) [30]. We also consider a case study in Alzheimer’s Disease (AD) using the IPECAD open-source model framework cross-validated to an existing Institute for Clinical and Economic Review model [31]. These case studies were chosen because the required information was publicly available, and they represented populations in which carer HRQoL is commonly considered in economic evaluation [4].

In each example, we assume that patients have one carer throughout their lifetime. We recognise that in some instances, multiple carers may be assumed or carers may only be included while the patient is aged under 18 years, or the number of carers may change over time. We make these simplifying assumptions to estimate the impact in a consistent manner and explore these additional factors in scenario analyses.

The input data for all examples are presented in Table 1. We additionally needed patient disutilities (see Sect. 2.2) so we estimated these by comparing the health state values to the best health state value without a carer disutility for SMA [30] and DMD (since the best health state in DMD still required a carer), and to the Health Survey for England expected EQ-5D-3L score for a 70-year-old male for AD [32]. For the utility approach we needed carer utilities, so for SMA and AD, we estimated carer utilities by combining the reported carer disutilities with expected EQ-5D-3L for a female aged 30 years (SMA) and 70 years (AD) [32]. The DMD source reported carer utilities as well as disutilities. We did not adjust for the impact of aging on HRQoL. The only additional data we used was the family effect of 0.123 [22].

**Table 1:** Input data for case studies

		Patient utility		Carer disutility	Life years: intervention	Life years: comparator
		Intervention	Comparator			
Duchenne Muscular Dystrophy (DMD) [29]	Ambulatory	0.932	0.617	− 0.070	17.607	11.572
	FVC > 50%	0.318	0.164	− 0.080	4.465	5.610
	FVC < 50%	0.318	0.164	− 0.140	7.170	5.122
	FVC < 30%	0.318	0.164	− 0.140	2.987	2.988
Spinal Muscular Atrophy (SMA)^a^ [30]	Assisted ventilation	0.190		− 0.080	0.190	2.180
	Not sitting	0.190		− 0.080	1.940	1.260
	Sits unassisted	0.600		− 0.030	12.000	0.000
	Walks unassisted	0.954		0.000	0.470	0.000
	Within a broad range of development	0.954		0.000	3.660	0.000
Alzheimer’s Disease (AD) [31]	MCI community	0.681		− 0.016	2.136	1.698
	Mild AD: community	0.631		− 0.022	1.779	1.549
	Moderate AD: community	0.491		− 0.039	0.790	0.941
	Severe AD: community	0.321		− 0.060	0.597	0.730
	MCI institution	0.681		− 0.016	0.169	0.132
	Mild AD: institution	0.631		− 0.022	0.197	0.164
	Moderate AD: institution	0.491		− 0.039	0.234	0.243
	Severe AD: institution	0.321		− 0.060	0.593	0.698

*FVC* forced vital capacity, *MCI* mild cognitive impairment

^a^Results for SMA are discounted, results for DMD and AD are undiscounted

### Calculating Family and Caregiving Effects

**Fig. 1:**
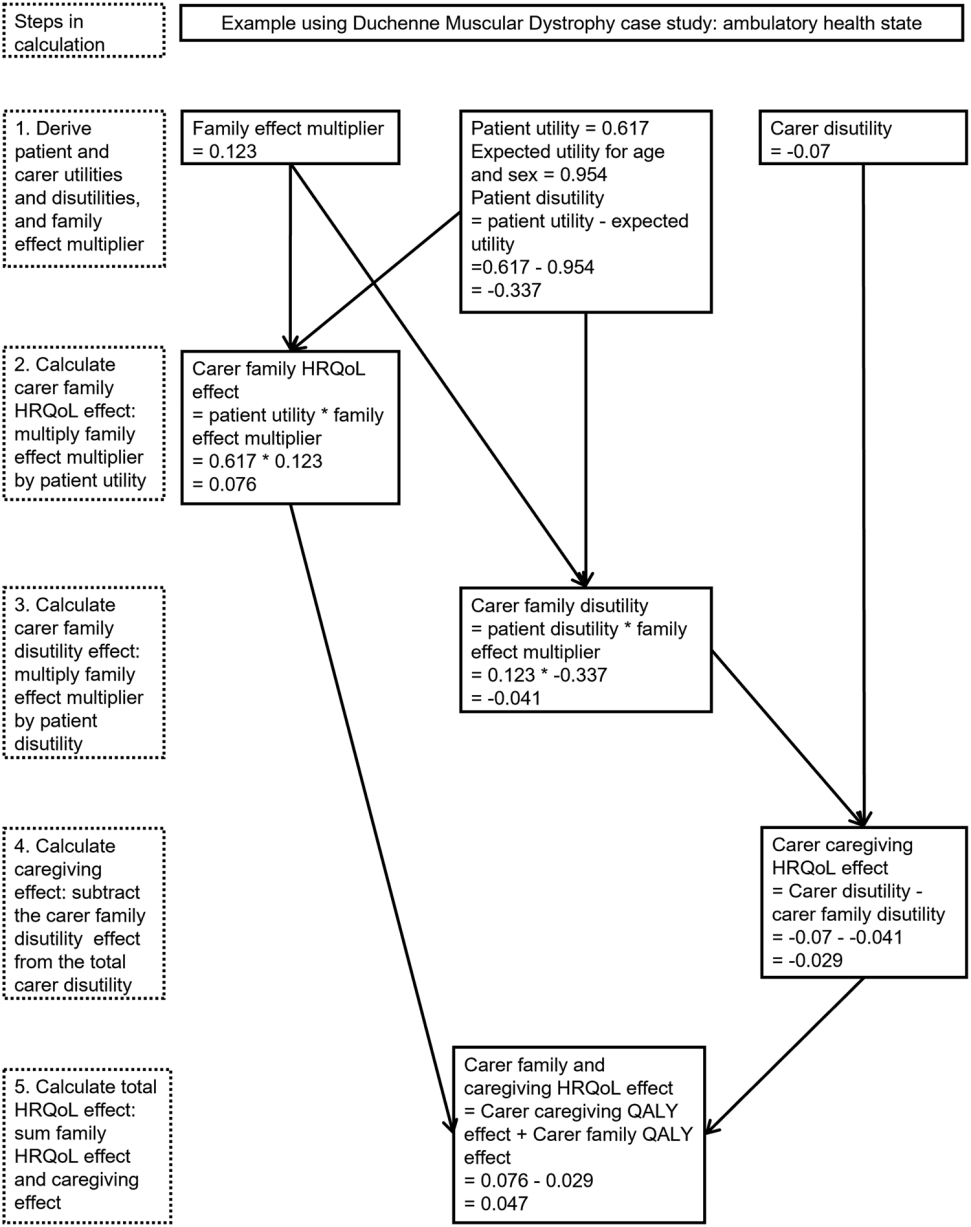
Calculating family and caregiving effects, with example for ambulatory health state in Duchenne Muscular Dystrophy

Figure 1 demonstrates how to calculate family and caregiving effects and use them to calculate the ‘family and caregiving’ QALY effect, for each pair of patient and carer (dis)utilities (for example, for each health state in a state-transition model). The example shown is for ambulatory health state on the comparator (best supportive care) for DMD. Here the family effect is positive, and the caregiving effect is negative, and the overall family and caregiving effect is positive.

### Results

Table 2 presents the patient QALYs and carer QALY effects from the three different methods, for all scenarios. Results are presented for the intervention, comparator, incremental, and the incremental carer QALY gain as a percentage of the incremental patient QALY gain to demonstrate its relative importance in calculating total incremental (patient and carer) QALYs.

**Table 2:** Results for three case studies

	Patient QALYs	Carer QALY effects		
		Carer utilities	Carer disutilities	Family and caregiving effect
Duchenne muscular dystrophy (DMD)				
Intervention	21.060	26.525	− 3.013	1.728
Comparator	9.390	20.828	− 2.391	0.573
Incremental	11.670	5.696	− 0.622	1.154
Carer QALY gain/patient QALY gain		49%	− 5%	10%
Spinal muscular atrophy (SMA)				
Intervention	11.545	15.977	− 0.723	1.612
Comparator	0.654	2.836	− 0.323	0.128
Incremental	10.891	13.142	− 0.399	1.484
Carer QALY gain/patient QALY gain		121%	− 4%	12%
Alzheimer’s disease (AD)				
Intervention	3.658	4.704	− 0.369	0.306
Comparator	3.315	4.429	− 0.378	0.260
Incremental	0.342	0.275	0.09	0.046
Carer QALY gain/patient QALY gain		80%	3%	13%

The SMA and DMD interventions lead to a substantial gain in patient QALYs owing at least in part to their anticipated survival benefit. The patient QALY gain for AD is much smaller, since the gain in patient life years is more modest (0.46 [31]). The carer QALY gains, in absolute terms, are therefore much higher for the SMA and DMD case studies, but the carer QALY gain relative to the patient QALY gain is similar in magnitude. This also explains why the carer disutilities approach leads to a QALY loss in the DMD and SMA case studies but not the AD case study: the anticipated substantial survival extension in DMD and SMA leads to the ‘carer QALY trap’ [9].

**Fig. 2:**
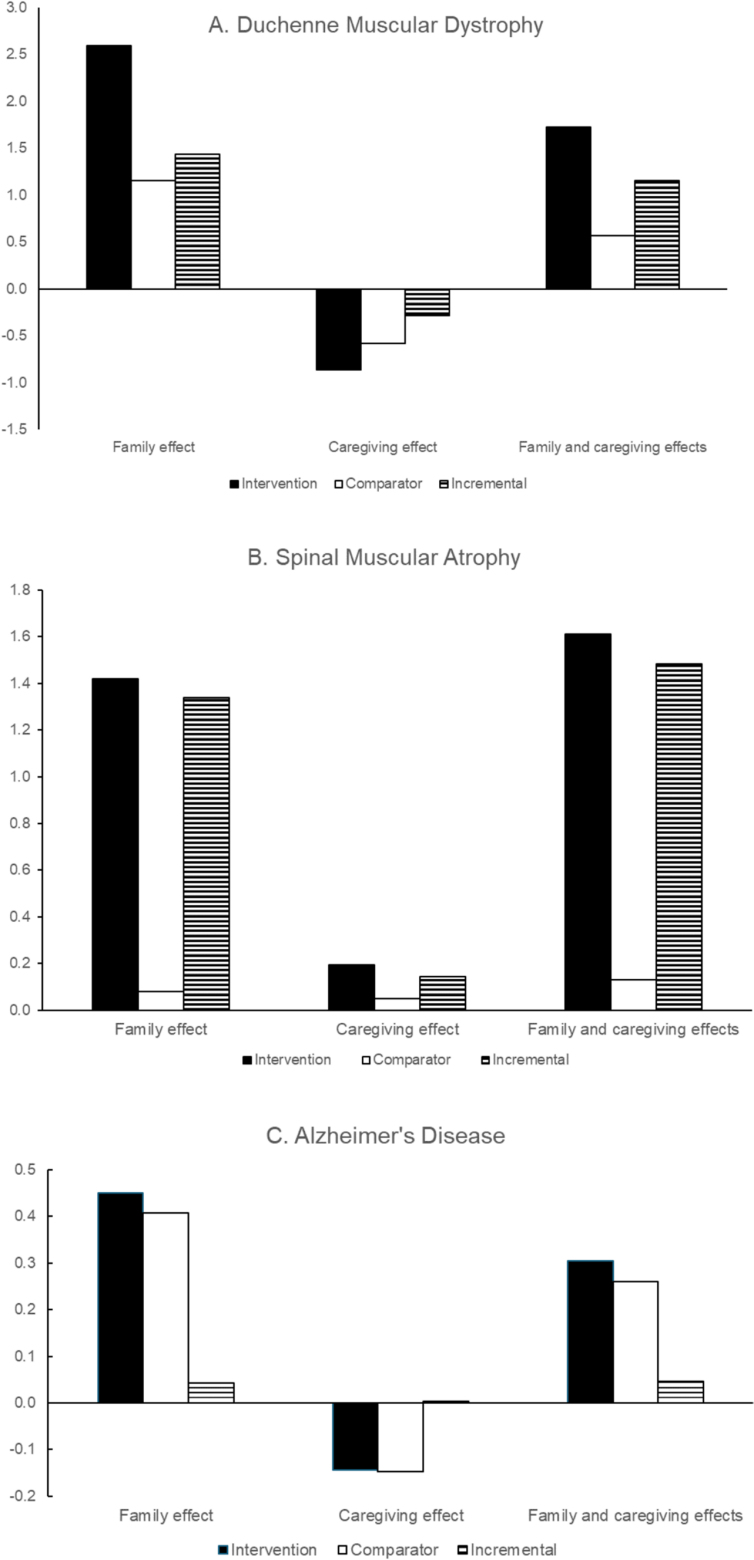
Family and caregiving effects for three case studies

Figure 2 presents the family and caregiving effects separately and combined for the three case studies. The family effect is always positive since all use the same source and all patient utilities are positive, as discussed in Section. 2.2, but the calculated caregiving effect differs.

As expected, the caregiving effect in DMD is negative. However, the relationship between patient and carer utilities in DMD is complex, since the company submission assumed that patient utility was treatment dependent, but carer disutility was treatment independent. Within the same health state, patients receiving the intervention are assumed to have a higher utility value than patients receiving the comparator, but carers are assumed to have the same utility regardless of the patients’ treatment. The carer family disutility (family effect multiplier multiplied by patient disutility) would therefore be larger for the comparator than the intervention. The carer disutility (sum of carer family disutility and caregiving effect) could then only be the same for intervention and comparator if the negative caregiving effect was larger for the intervention than the comparator. We propose that this is unlikely to be intended or plausible (unless the intervention somehow increases the caregiver burden), and so in our analysis we assumed the same caregiving effect for intervention and comparator, which led to a smaller total carer disutility for the intervention.

Somewhat surprisingly, the caregiving effect in the SMA case study is positive (see positive caregiving effect bar in Fig. 2): this arises because of the relative sizes of the patient and carer utilities. In the ‘sits unassisted’ health state, the patient utility is 0.60, and the carer disutility is − 0.03. With a family effect multiplier of 0.123, and assuming the patient disutility is − 0.354 (relative to the utility of 0.954 in the best health state), we would expect the carer disutility due solely to the family effect to be − 0.044 (0.123 * − 0.354). Since the total carer disutility is less than this, this implies that the caregiving effect must be positive: providing care to a child with SMA alleviates some of the negative HRQoL impact of living with a child with SMA. By comparison, in AD disease in the ‘mild AD’ health state, the patient utility is 0.631 and the carer disutility is −0.05, somewhat larger than the disutility in SMA and larger than the disutility due to the family effect, leading to a negative caregiving effect.

The carer QALYs gained due to the family effect are much larger than the carer QALYs lost or gained through the caregiving effect in all the case studies. This is because the disutility for carers is fairly close to the family effect itself, so the additional effect of caregiving is relatively small. If the family effect were smaller or the total caregiver disutility were bigger, the relative effect of the caregiving effect would be larger. This is explored in scenario analysis in Table 3: with a larger family effect (0.163 [14] versus 0.123), the carer QALY effects increase for intervention and comparator, and the incremental carer QALY effect increases. With a smaller family effect (0.0642 [20] versus 0.123), the caregiving effect becomes bigger and negative for all interventions and comparators (including SMA), and the combined family and caregiving effect decreases. The size of carer QALY gain relative to patient QALY decreases as the family effect decreases.

**Table 3:** Scenario analyses using different family effect sizes

	Family and caregiving effect	
	Family effect = 0.16	Family effect = 0.0642
*Duchenne muscular dystrophy (DMD)*		
Intervention	3.154	− 0.539
Comparator	1.465	− 0.844
Incremental	1.689	0.305
Carer QALY gain/patient QALY gain	14%	3%
*Spinal muscular atrophy (SMA)*		
Intervention	2.257	0.588
Comparator	0.250	− 0.065
Incremental	2.007	0.652
Carer QALY gain/patient QALY gain	18%	6%
*Alzheimer’s disease (AD)*		
Intervention	0.508	− 0.015
Comparator	0.452	− 0.045
Incremental	0.057	0.028
Carer QALY gain/patient QALY gain	17%	8%

## Discussion and Conclusion

Separating carer QALY effects into the family effect and the caregiving effect allows us to consider both the positive spillover of extending patient survival and/or improving patient HRQoL, and the potentially negative (but in some cases positive) spillovers of the HRQoL burden of providing care. This removes the need for extreme and unrealistic implications of either the carer utility or disutility approaches typically used in HTA, where caring has to have a wholly positive or wholly negative impact on HRQoL. Viewing carer (dis)utilities in the context of the family and caregiving effects can also provide context for how realistic they are – our SMA example highlights that the carer disutilities may be underestimated. We demonstrated our approach using the disaggregated results from economic models, but ideally, we would recommend that the caregiving and family effects are included within the model calculations.

Our case studies assumed each patient had one carer, but it is possible to extend this approach to consider additional carers. The additional carers could have smaller family effect, estimated for example using geometric/arithmetic progression as in an example in meningitis [15]. It may also be appropriate to assume the caregiving effect reduces or becomes zero for family members who care about the patient, but do not actively provide care.

We demonstrated that our approach can be applied in any economic evaluation where the inclusion of carers is relevant, if the patient and carer (dis)utilities are known, and an estimate of the family effect multiplier is available (from a specific analysis or the wider literature) . There are two key limitations with this: whether the family effect multiplier can be assumed to be transferable across populations, and whether the patient and carer utility data can be combined in this way. We suggest that the current evidence indicates that the family effect is generalisable, but is limited to populations of adults, or children with acute health conditions. Future research should consider whether the family effect multiplier is the same for chronic paediatric health conditions such as DMD and SMA. It may also be influenced by other factors such as the relationship between the patient and carer, presence of other household members and their roles in caregiving, as well as any support received outside the household. Where analysts are assuming the family effect multiplier can be transferred from other conditions, scenario analysis with alternative values could help to address this uncertainty.

Our approach highlights the challenges in combining patient and carer data. The issues of measuring carer HRQoL are well described elsewhere [33] but would be ideally collected alongside patient HRQoL to allow analysis of dyads and estimation of the specific family effect. Our finding of a positive caregiving effect in SMA may be in part owing to this: the patient and carer (dis)utilities were sourced from separate studies, the carer utilities were from a population caring for children with a different indication, and the two studies used different measures [34, 35]. There is a separate challenge in collecting and measuring patient HRQoL data in the specific populations where carers are required, for example in paediatric populations [36, 37] or populations with cognitive impairment [38, 39] – while this challenge is not specific to our approach, it does compound the issue of combining patient and carer data.

Policymakers may question whether it is appropriate to include both the family effect and the caregiving effect in economic evaluation when all patients across health conditions may be expected to have family members who care about them, even if they do not actively care for them. It is unclear whether data supports the existence of a family effect for non-caring dyads: in the Bobinac et al. analyses all carers were actively providing care [20, 21], but in the Pennington et al. analysis carers were not necessarily caring at all time points [22]. If policymakers do determine that the family effect applies in all conditions regardless of the need for carers, then it may be appropriate to either only include the caregiving effect in economic evaluations that require unpaid carers, or to include the family effect in all economic evaluations. In the latter scenario, cost-effectiveness thresholds should also be reduced to reflect the family effect in the opportunity cost, for example, assuming the family effect multiplier of 0.123, the £20,000/QALY threshold would be divided by 1.123 to become £17,809/QALY, and the £30,000/QALY threshold would become £26,714/QALY. However, the size of the family effect multiplier may vary across conditions, with 0.123 as the average and some conditions having much higher (and lower) family effects.

By separately considering the family and caregiving effects on carer QALYs, economic evaluations can reflect the positive and negative spillovers associated with extending patient survival and improving patient HRQoL. Our method uses data already routinely included in economic evaluations that include carers, in combination with published estimates for the family effect. Future economic evaluations can use our method and data to include carers, and future research should seek to estimate the family effect in different populations.

## Declarations

### Data and/or Code availability

All data used in this manuscript are available in the public domain in the sources referenced.
